# Photoluminescence Quenching and Enhanced Optical Conductivity of P3HT-Derived Ho^3+^-Doped ZnO Nanostructures

**DOI:** 10.1186/s11671-016-1630-3

**Published:** 2016-09-20

**Authors:** Guy L. Kabongo, Pontsho S. Mbule, Gugu H. Mhlongo, Bakang M. Mothudi, Kenneth T. Hillie, Mokhotjwa S. Dhlamini

**Affiliations:** 1 Department of Physics, University of South Africa, PO Box 392, 0003 Pretoria, South Africa; 2 CSIR-National Centre for Nano-Structured Materials, PO Box 395, 0001 Pretoria, South Africa; 3 Département de Physique, Université Pédagogique Nationale, 8815 Kinshasa, République Démocratique du Congo; 4 Department of Physics, University of Free State, Bloemfontein, 9300 South Africa

**Keywords:** P3HT-ZnO:Ho^3+^, Charge transfer, UV-Vis absorption, PL quenching, XPS

## Abstract

In this article, we demonstrate the surface effect and optoelectronic properties of holmium (Ho^3+^)-doped ZnO in P3HT polymer nanocomposite. We incorporated ZnO:Ho^3+^ (0.5 mol% Ho) nanostructures in the pristine P3HT-conjugated polymer and systematically studied the effect of the nanostructures on the optical characteristics. Detailed UV-Vis spectroscopy analysis revealed enhanced absorption coefficient and optical conductivity in the P3HT-ZnO:Ho^3+^ film as compared to the pristine P3HT. Moreover, the obtained photoluminescence (PL) results established the improvement of exciton dissociation as a result of ZnO:Ho^3+^ nanostructures inclusion. The occurrence of PL quenching is the result of enhanced charge transfer due to ZnO:Ho^3+^ nanostructures in the polymer, whereas energy transfer from ZnO:Ho^3+^ to P3HT was verified. Overall, the current investigation revealed a systematic tailoring of the optoelectronic properties of pristine P3HT after inclusion of ZnO:Ho^3+^ nanostructures, thus opening brilliant perspectives for applications in various optoelectronic devices.

## Background

Organic-inorganic semiconductors are the main focus of tremendous research activities nowadays due to their promising prospective in optoelectronic device applications, particularly related to their wide photophysical potentialities [1, 2]. It is well known that the fundamental properties of P3HT-conjugated polymers can be tailored intentionally through various designs and structural modification in order to achieve optimal efficiencies [3, 4]. Pristine P3HT has been widely applied in the fabrication of optoelectronic devices but mostly in bulk heterojunction organic photovoltaic devices due to its low bandgap and especially its high absorption coefficient (order of 10^5^ cm^−1^) in the visible [3, 5–10]. It is however of crucial importance to probe and elucidate the mechanisms underlying the versatile properties of the modified organic polymer semiconductor materials. Moreover, in optoelectronic applications, one of the major drawbacks of organic semiconductors is their low charge mobility. In order to overcome this, we have proposed a hybrid heterostructure based on pristine P3HT containing inorganic ZnO:Ho^3+^ nanostructures, which are aimed at enhancing its optoelectronic properties owing to the advantages offered by the high electron mobility of ZnO. It is also important to note that ZnO nanoparticles have been previously found to enhance carrier mobility in P3HT [11].

There are plenty of works in the literature related to hybrid-based polymer heterostructures containing inorganic nanostructures [3, 12–17]. Several research groups which worked on P3HT-ZnO heterostructures devoted their focus on various aspects such as power conversion efficiency (PCE) enhancement [7, 18], morphology, structural properties, exciton generation, thermal properties, photoexcitation, and charge dynamic [19–21]. However, to our best knowledge, there is no report about optical conductivity of P3HT-ZnO:Ho^3+^ heterostructures in the literature. Moreover, there is no available report found in the literature on photoluminescence quenching of P3HT-ZnO:Ho^3+^ heterostructure. Owing to its attractive 4*f-*4*f* intra-ionic transitions, holmium is expected to tune the optical properties of ZnO and thus leads to the P3HT-ZnO:Ho^3+^ system exhibiting excited overall performance [22]. Previous studies have shown that materials containing holmium displayed rich absorbing characteristics in the visible range of the electromagnetic spectrum [23, 24]. Furthermore, Lian et al. [25] suggested that through up-conversion processes, Ho^3+^-based structures may absorb ultra-broadband near-infrared (UBB-NIR) photons and convert them to visible NIR photons above the bandgap of the host matrix.

In the current paper, we investigated the effect of Ho^3+^ on the optical and surface properties of P3HT-based heterostructure which is the subject of extensive investigation for application in solar cells and other optoelectronic devices. The main motivation of the study was to demonstrate a novel approach to achieve efficient interfacial charge transfer in P3HT-ZnO heterostructure. The incorporation of Ho^3+^ dopants leads to the enhancement of the crystallinity of ZnO and further improved the charge mobility of P3HT-ZnO:Ho^3+^ as compared to P3HT-ZnO heterostructure which can be understood through its optical conductivity. Such heterostructures are of particular interest as absorbing layers in organic-inorganic bulk heterojunction solar cells. Finally, to our best knowledge, there is no report in the literature which describes the photoluminescence quenching in P3HT-ZnO heterostructure owing to the presence of Ho^3+^ ions in the crystal lattice of ZnO.

## Methods

The precursors were all used as received from Sigma-Aldrich (Germany). ZnO:Ho^3+^ were synthesized by a sol-gel method following the same procedure as reported in our previous works [26, 27]. The solution of sodium hydroxide dissolved in ethanol was prepared separately, then cooled in ice water and added drop-wise judiciously to the ethanol suspension of Zn^2+^ ions. For the preparation of Ho^3+^-doped ZnO samples with different concentrations of Ho^3+^ (0.5 mol%), the ethanol solution of holmium nitrate pentahydrate was added into the hydrolyzed Zn^2+^ solution prepared following the above route. The obtained clear solution was kept at room temperature for 24 h and then washed several times in a mixture of ethanol and heptane (1:2 molar ratio) to eliminate unreacted Na^+^ and CH3COO^−^ ions. The resulting precipitates were then re-dispersed in ethanol and/or dried at 200 °C for 2 h.

Moreover, P3HT-ZnO:Ho heterostructures films were prepared via a direct mixing solution drop-casting method of ZnO and pristine P3HT assisted by ultrasonication [28].

The optical microscopy images were obtained on a KEYENCE (VK-X250/X150/X120) 3D Laser scanning confocal microscope. The room temperature (RT) UV-Vis absorption spectra were collected using a Perkin-Elmer Lambda 1050 UV/Vis/NIR spectrophotometer equipped with integrated sphere. Photoluminescence (PL) spectra were collected at RT using a Jobin Yvon Fluorolog 3 spectrofluorometer. The X-ray photoelectron spectroscopy (XPS) core levels were carried out using a PHI 5000 VersaProbe-Scanning ESCA Microprobe. Raman scattering were collected using a Horiba Jobin Yvon HR800 Raman spectrometer equipped with a visible microscope with a 514-nm-excitation Ar^+^ laser with a spectral resolution of 0.4 cm^−1^ at RT.

## Results and Discussion

### TEM and XRD Analysis of Un-Doped and Ho^3+^-Doped ZnO

**Fig. 1:**
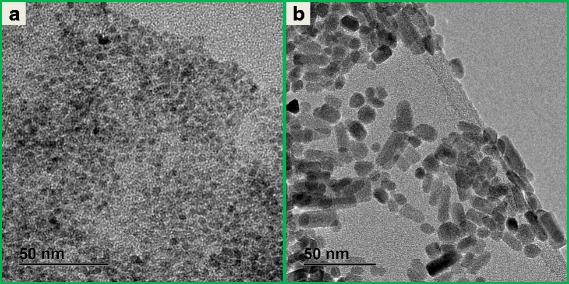
TEM images for **a** un-doped and **b** Ho^3+^-doped ZnO

TEM measurements were undertaken carefully on both un-doped and Ho^3+^-doped ZnO samples (see Fig. 1). The measurements revealed that the nanostructures were evenly distributed and were highly crystalline. However, a peculiar phenomenon based on the mutation of morphology was observed and was attributed to doping with Ho^3+^ ions, more precisely due to the growth of nanoparticles in solution through a diffusion-limited Ostwald ripening process known to be the most predominantly growth mechanism so far [29]. However, further investigations are required in order to effectively elucidate on the observed mechanism of morphology mutation. It is however suggested that studies on colloidal nanocrystals growth under TEM using the so-called liquid cell electron microscopy [30, 31] should be performed. Such study has been undertaken previously but not quite intensively, especially on the ZnO nanoparticles growth [32]. Furthermore, TEM analysis revealed that the particle diameter of both un-doped and Ho^3+^-doped ZnO was below 10 nm.

**Fig. 2:**
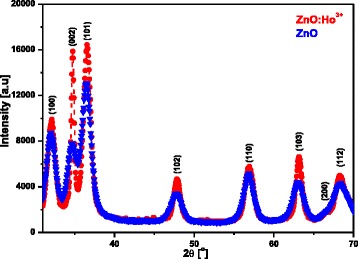
XRD patterns for un-doped ZnO and Ho^3+^-doped ZnO dried at 200 °C

Figure 2 illustrates the X-ray diffraction (XRD) profiles of the ZnO and ZnO:Ho^3+^. The analysis revealed that the as-synthesized nanocrystals ranging between 4 and 8 nm in diameter were highly crystalline and exhibited the hexagonal wurtzite structure (space group *P*6_3_*mc*) indexed to JCPDS card # 36-1451. Furthermore, no second phase originating from Ho_2_O_3_ was observed in the Ho^3+^-doped ZnO samples, which confirms that the dopants successfully substituted the Zn^2+^ ions within the ZnO lattice structure. Scherrer’s equation was employed to estimate the crystallite size [26, 27, 33]

1 $$ D=\frac{k\lambda }{\beta\  \cos \uptheta}, $$

where *D,* λ*,* β*, Ө*, and *k* are the crystallite size, the wavelength of the incident X-ray CuK radiation (0.1514 nm), the full width at half maximum (FWHM), the diffracting angle, and a numerical constant (0.94), respectively. The result revealed that Ho^3+^ doping improved the crystallinity of ZnO as confirmed by the enhanced intensity of the diffraction peaks and the crystallinity increased with Ho^3+^.

### UV-Vis Absorption and Optical Microscopy Analysis

**Fig. 3:**
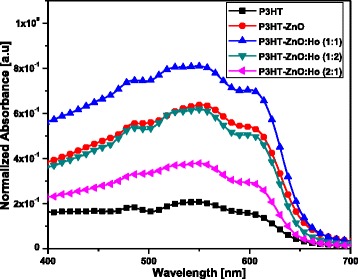
Absorption spectra of pristine P3HT, P3HT-ZnO, and P3HT-ZnO:Ho^3+^ films

Figure 3 shows normalized absorption spectra of pristine P3HT, P3HT-ZnO, and P3HT-ZnO:Ho^3+^ with several P3HT to ZnO:Ho^3+^ mass ratio (1:1, 1:2, 2:1). An obvious improved absorbance for pristine P3HT was observed from the film containing a blend of P3HT and ZnO:Ho^3+^ nanostructures (1:1 mass ratio). It has been reported elsewhere that such improvement in absorbance intensity leads to enhanced efficiency of polymer solar cells [17]. In the current study, all samples exhibited the common vibronic modes of the excited electronic state absorption peaks centered at about 526 nm (2.38 eV), 548 nm (2.26 eV), and 602 nm (2.06 eV) assigned to A_0-2_, A_0-1_ intra-chain excitation and A_0-0_ transition (π-π inter-chain interaction), respectively [34–36]. In addition, an isosbestic peak located at about 479 nm (2.59 eV) was detected, which denote the presence of two different states in the polymer [36]. The isosbestic peak may be explained by a transition of completely dissolved polymer chains into aggregated stacks without intermediate states [36]. It was noted that all spectra exhibited an absorption band-edge at about 650 nm. Furthermore, the redshift observed in the band-edge of P3HT-ZnO:Ho^3+^ (1:1) film as compared to the P3HT film result in an enhanced inter-chain interaction in semicrystaline P3HT leading to effective ground state interaction between the polymer and ZnO:Ho^3+^ nanostructures [3, 17]. In fact, the nanostructures induced an improvement of the structure ordering of the hybrid heterostructure [7]. The observed redshift, which involve enhanced conjugation length, definitely implies an enhancement of the light absorption ability of P3HT polymer with inclusion of ZnO:Ho^3+^ nanostructures [1].

Spano and co-workers have established the correlation between the absorption lineshape and the weakly interacting H-aggregates states [37]. Assuming that the Huang-Rhys factor is equivalent to unity [38], the free-exciton bandwidth of the aggregates (*W*) has been calculated using the equation below [37–40]:

2 $$ {I}_{0-0}{I}_{0-1}={\left(\frac{1-0.24W/{E}_p}{1+0.073W/{E}_p}\right)}^2, $$

where *I*_0 − 0_ and *I*_0 − 1_ are the magnitudes of A_0–0_ and A_0–1_ transitions, respectively. *E*_*p*_ = 180 meV is the effective energy of the main intramolecular vibrational modes coupled to the electronic transition [41]. The average values obtained for (*W*) were 420, 389, and 378 meV for P3HT, P3HT-ZnO, and P3HT-ZnO:Ho^3+^, respectively. This result implies an increase in conjugation length and chain ordering with the inclusion of ZnO:Ho^3+^ nanostructures in P3HT [42–45].

The UV-Vis spectra collected in the current study served to analyze the refractive index, dielectric constant, and optical conductivity of the samples (see Table 1). Optical conductivity is a key parameter to evaluate optoelectronic materials and has been previously employed to probe carrier dynamic in organic semiconductors [46]. It is related to the electrical conductivity within a variable electric field. Prior to the investigation of optical conductivity, the absorption coefficient was calculated following the Beer-Lambert law [47]:

**Table 1:** Optical parameters of P3HT and P3HT-ZnO:Ho^3+^ films at 548 nm

Sample	*α*	*K*	*n*	σ [S^−1^]	*ɛ*_i_	*ɛ*_r_
P3HT	0.18	7.72	1.69	0.72 × 10^7^	26	−57
P3HT-ZnO:Ho^3+^	0.22	9.55	3.17	1.66 × 10^7^	61	−81

3 $$ \log \left(\frac{I_0}{I_t}\right)=2.303\times A=\alpha \times t, $$

where *I*_0_ and *I*_*t*_ are the intensities of the incident and transmitted light beam, respectively. Moreover, (*A*) and (*t*) parameters are the optical absorbance and thickness of the film, respectively.

Table 1 presents the extinction coefficient of the samples, which is the measure of the light scattering loss and absorption per unit distance of the penetration medium. Moreover, the extinction coefficient is proportional to the absorption coefficient as presented in the following equation [48]:

4 $$ K=\frac{\alpha \lambda }{4\pi }, $$

where *α* and *λ* are the absorption coefficient and the wavelength of the photon, respectively.

The refractive index was calculated using the equation below [49]:

5 $$ n={\left(\frac{1}{T} - 1\right)}^{\frac{1}{2}}+\frac{1}{T}, $$

where *T* is the percent transmission coefficient.

The dielectric constant composed of a real (*ε*_r_) and an imaginary (*ε*_i_) component was obtained as a function of “*K*” and “*n”* as shown below [50]:

6 $$ {\varepsilon}_{\mathrm{r}}={n}^2-{K}^2 $$

7 $$ {\varepsilon}_{\mathrm{i}}=2nK. $$

Finally, the optical conductivity was obtained as described below [50, 51]:

8 $$ \sigma =\frac{\alpha nc}{4\pi } $$

where α, *n*, and *c* are the absorption coefficient, refractive index, and celerity of light in the vacuum, respectively.

**Fig. 4:**
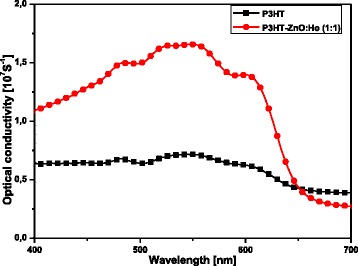
Optical conductivity spectra of pristine P3HT and P3HT-ZnO:Ho^3+^ films

At 548 nm, the optical conductivity was found to increase by the order of 2.3 with the inclusion of ZnO:Ho^3+^ nanocrystals in pristine P3HT (see Fig. 4). The dependence of the optical conductivity with the absorption coefficient complies very well with previous studies [50]. The variation observed in the optical conductivity upon ZnO:Ho^3+^ nanostructure inclusion can be associated to improved electronic transport properties in the heterostructure material. More interestingly, larger dielectric constant is beneficial to improving power conversion efficiency of photovoltaic devices [52].

**Fig. 5:**
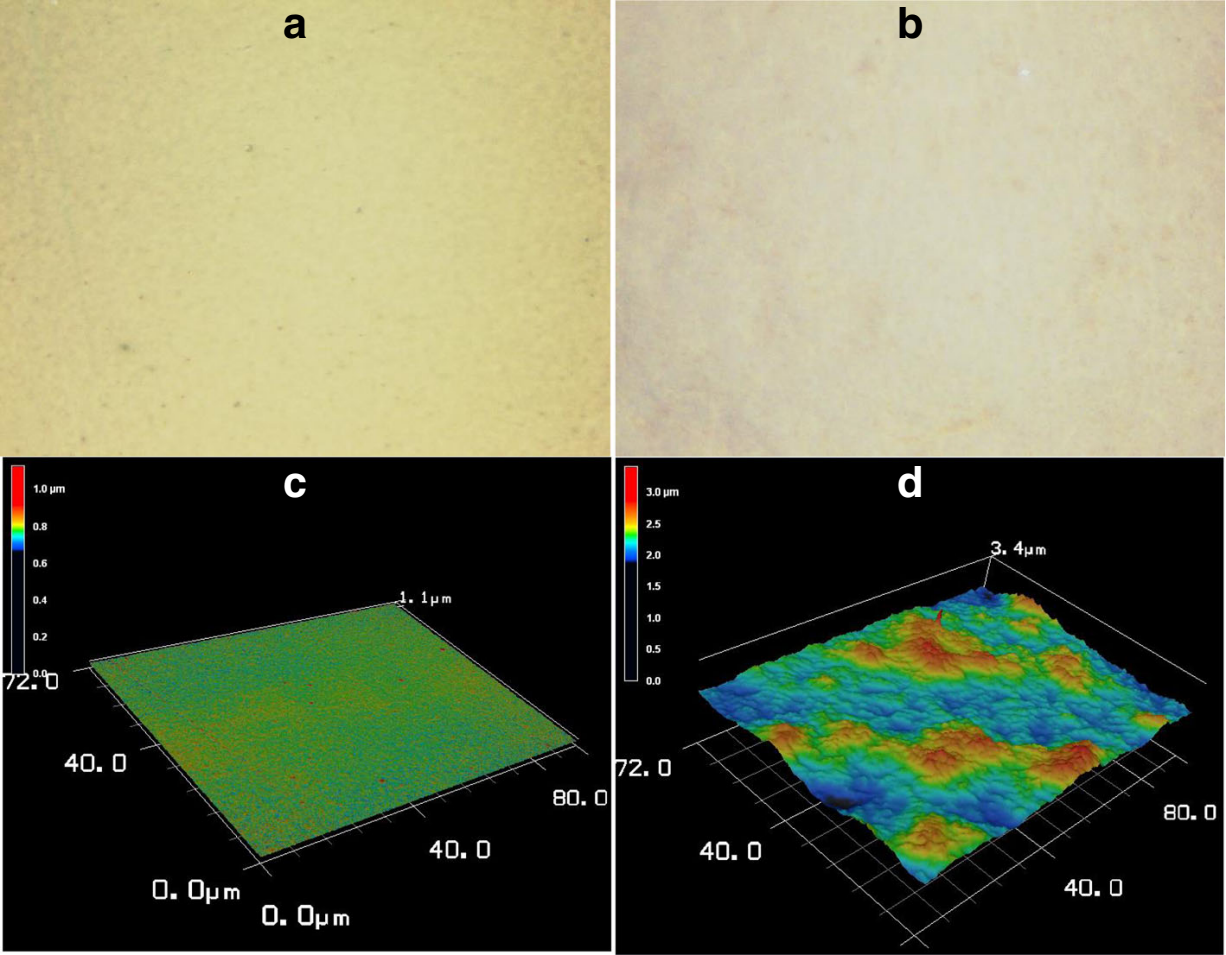
72 × 80 μm^2^ laser confocal optical and 3D micrographs of (*left panel* **a**, **c**) pristine P3HT and (*right panel* **b**, **d**) P3HT-ZnO:Ho^3+^ (1:1) films

To probe the effect of ZnO:Ho^3+^ nanostructures on the surface morphology of pristine P3HT film, we collected 3D optical micrographs of both P3HT and P3HT-ZnO:Ho^3+^ films as shown in Fig. 5. The inclusion of ZnO:Ho^3+^ nanostructures in the P3HT polymer greatly altered the morphology of the donor (P3HT)-acceptor (ZnO:Ho^3+^) interface of the hybrid heterostructure [53, 54]. The images clearly demonstrated that the addition of ZnO:Ho^3+^ nanostructures has increased the root-mean-square surface roughness (SSR) of the P3HT-ZnO:Ho^3+^ film (140.7 nm) as compared to pristine P3HT film (30.4 nm), which exhibited a smooth and blemished surface (Fig. 5a, c). More importantly, the P3HT-ZnO:Ho^3+^ heterostructure (see Figs. 5b and 2d) exhibited aggregation of ZnO:Ho^3+^ nanostructures on the surface of the film and a higher coverage of the substrate with a height value of 3.4 μm as compared to the pristine P3HT film which exhibited a height of 1.1 μm. The observed increase of the surface roughness of the hybrid heterostructure film is in agreement with enhanced light absorption (see UV-Vis absorption results).

### Photoluminescence Analysis

**Fig. 6:**
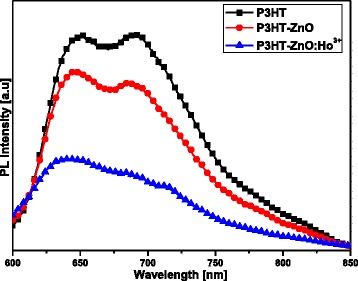
Emission spectra of pristine P3HT, P3HT-ZnO, and P3HT-ZnO:Ho^3+^ (1:1) films

The photoluminescence spectra (Fig. 6) of the samples were obtained through 548-nm (2.26 eV) photoexcitation; this energy correspond to the strongest vibronic state in the absorption spectra (see Fig. 3). It was found that the emission spectra were dominated by P3HT, while the ZnO emission was suppressed due to the energy transfer from ZnO to P3HT [55]. The origin of this emission is still controversial and still need lot of studies in order to effectively establish its deep origin. The spectra contains two peaks at about 649 nm (1.91 eV) and 692 nm (1.79 eV), which are respectively attributed to the (0-0) and (0-1) transitions of H-aggregates in the regio-regular P3HT films; the obtained spectra revealed a quenching of the P3HT emission which is beneficial for photovoltaic solar cells [41, 56, 57]. Interestingly, the blueshift of the main emission peak (0-0 transition) of P3HT-ZnO (*λ*_em_ = 647 nm, 1.92 eV) and further the P3HT-ZnO:Ho^3+^ (*λ*_em_ = 639 nm, 1.94 eV) as compared to the pristine P3HT film was assigned to the alteration of P3HT crystalline order [55, 58]. Moreover, based on the abundance of the report found in the literature, and in the light of their successful luminescence studies so far, photoluminescence quenching appear to be a key indication of the measure of success of exciton dissociation in P3HT-based materials [15]. The spectra recorded in the current study exhibited two well-defined peaks in the range of 600–800 nm which either agrees well with the previous report [15]. Moreover, the quenching of P3HT emission denotes effective enhanced exciton dissociation at the interface of ZnO nanostructures and P3HT host matrix. It was found that the quenching was further important in the case of P3HT-ZnO:Ho^3+^ heterostructure; this is possibly due to the occurrence of more quenching centers within the polymer resulting from holmium doping [59]. However, further transient absorption spectroscopy study is needed to elucidate more on this process. Generally, two types of PL quenching phenomena may occur in polymers: static and dynamic quenching [60, 61]. The pillar for this process to take place is the interaction between a fluorophore (P3HT) and a quencher (ZnO:Ho^3+^). In the process of static quenching, a non-fluorescent complex forms between a fluorophore and a quencher. The formation of this complex is independent of the population of the excited state [61]. On the other hand, the dynamic quenching also known as collisional quenching is the result of a diffusion of the quencher to the fluorophore during the lifetime of the excited state. The diffusion will result in the return of the fluorophore to the ground state without emission of a photon [61]. It is worth noting that the collisional quenching is optimally described by the Stern-Volmer law [61]. The quantitative PL quenching analysis revealed that the quenching rate (∆PL/PL_0_; ∆PL ≈ PL_0_ − PL_f_) was about 0.57. Overall, the degree of quenching of the polymer photoluminescence is a good indication of efficient charge transfer induced by the acceptor (ZnO:Ho^3+^) dispersed in the donor host matrix (P3HT).

### X-ray Photoelectron Spectroscopy Analysis

**Fig. 7:**
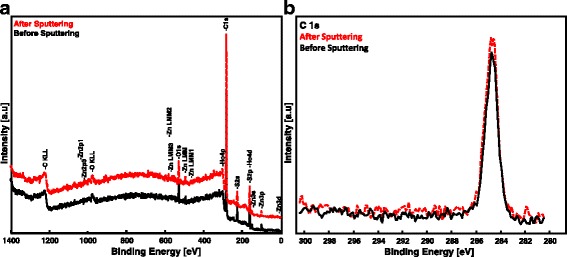
XPS **a** wide survey scan spectra and **b** C 1*s* core level of P3HT-ZnO:Ho^3+^ film before and after sputtering

**Fig. 8:**
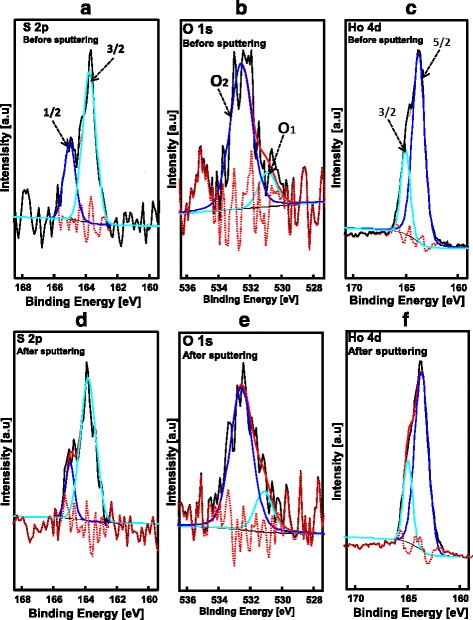
XPS S 2*p*, O 1*s*, and Ho 4*d* core levels of P3HT-ZnO:Ho^3+^ film **a**–**c** before and **d**–**f** after Ar^+^ sputtering

Surface state study and elemental analysis of the P3HT-ZnO:Ho^3+^ (1:1) film before and after Ar^+^ sputtering were undertaken using X-ray photoelectron spectroscopy. Prior to analysis, the binding energy (BE) calibration by means of C 1*s* peak (BE = 284.8 eV) was applied. Carbon, sulfur, oxygen, zinc, and holmium distribution in P3HT-ZnO:Ho^3+^ film were detected and analyzed (see Table 2). Figure 7a shows the wide survey scan spectra of P3HT-ZnO:Ho^3+^ film in which the two significant peaks at about 284 and 530 eV were identified to arise from C 1*s* and O 1*s* core levels, respectively. The lower BE region was found to be dominated by two peaks at about 227 and 163 eV from P3HT originating from S 2*s* and S 2*p* core levels. The absence of shake-up peak on the higher BE region of the high-resolution spectra of C 1*s* core level (Fig. 7b) indicates that the conjugation lengths of the conjugated *π* system in P3HT-based heterostructure remained unbroken after inclusion of ZnO:Ho^3+^ nanostructures [51, 62]. High-resolution measurements were performed on C 1*s*, S 2*p*, O 1*s*, and Ho 4*d* before and after Ar^+^ ion sputtering as shown in Fig. 8. The S 2*p* core level centered at about BE = 164 eV was de-convoluted to give rise to two components; the lower BE component at about 163 eV is related to the interaction between P3HT and ZnO (Fig. 8a). However, the higher BE component at about 165 eV is due to S–C bonding in P3HT [51, 53–56, 58–65]. Moreover, the spin-orbit splitting (∆) of the S 2*p* core level spectra was found to be 1.24 and 1.15 eV before and after Ar^+^ ion sputtering, respectively. The Ar^+^ sputtering was found to consolidate the interaction of P3HT and ZnO (Fig. 8d) as a result of the reduced concentration of the contaminant (see Table 2). The value of the spin-orbit splitting obtained after Ar^+^ sputter cleaning was found to comply well with the literature [66], reason being the removal of surface contaminant. Additionally, the de-convoluted high-resolution O 1*s* core level spectra depicted in Fig. 8b resulted in two bands: the lower BE = 530 eV is assigned to oxygen bonded with zinc and the higher BE band located at about 532 eV resulting from oxygen impurities incorporated in P3HT [67–69]. It is however important to note that the Ar^+^ ion sputtering causes the reduction of oxygen impurities concentration in P3HT (Fig. 8e). Finally, Fig. 8c, f presents the Ho 4*d* core level spectra of the sample before and after Ar^+^ sputtering, respectively. The peak was de-convoluted to give rise to a doublet bands located at BE = 163 and 165 eV assigned to Ho 4*d*_5/2_ and Ho 4*d*_3/2_, respectively [70].

**Table 2:** XPS core level positions of P3HT-ZnO:Ho^3+^ film before and after Ar^+^ sputtering

Core level [eV], area [%]	C 1*s*	O 1*s*		S 2*p*		Ho 4*d*	
		O_2_	O_1_	1/2	3/2	3/2	5/2
Before Ar^+^ sputtering	284.8	532.55	530.88	165.00	163.76	165.01	163.76
		(84.81 %)	(15.19 %)	(30.23 %)	(69.77 %)	–	
After Ar^+^ sputtering	284.8	532.55	531.11	164.95	163.80	164.95	163.66
		(80.96 %)	(19.04 %)	(18.08 %)	(81.92 %)	–

### Raman Spectroscopy Analysis

**Fig. 9:**
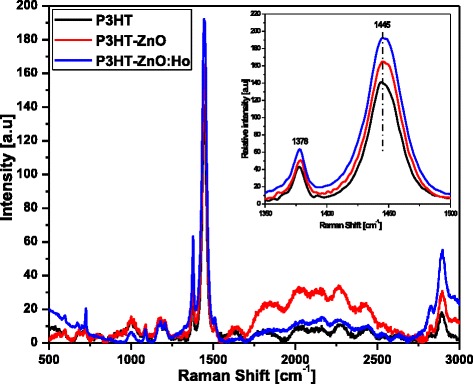
Raman scattering spectra of pristine P3HT, P3HT-ZnO, and P3HT-ZnO:Ho^3+^ (1:1) films

Further details about the spectroscopic properties were obtained by the analysis of the Raman scattering of pristine P3HT, P3HT-ZnO, and P3HT-ZnO:Ho^3+^ heterostructures as shown in Fig. 9. The most common Raman shift features of P3HT were identified to be active at 1445.36, 1378.22, and 726.55 cm^−1^, which were assigned to C_α_=C_β_ symmetric stretching mode, C_β_–C_β_ skeletal stretching mode, and to antisymmetric C_α_–S–C_α_ ring skeleton in-plane distortion in the thiophene ring of P3HT, respectively [71, 72]. The other less intense peaks falling in the range of 999.58, 1088.52, 1178.79, and 1514.96 cm^−1^ are assignable to C_β_–C_alkyl_ stretching mode, C–H bending mode, C_α_–C_α_ symmetric stretching mode, and C_α_=C_β_ antisymmetric stretch mode [71–74]. The peak located at higher Raman shift at about 2901.2 cm^−1^ was assigned to C–H antisymmetric stretching mode [34]. Furthermore, the blueshift observed in the doped sample could be the result of the interaction taking place between pristine P3HT and ZnO. Finally, as shown in the magnified Raman spectra in the region of 1350–1500 cm^−1^ (see inset Fig. 9), the intensity of the scattering of the P3HT-ZnO:Ho^3+^ film is found to increase as compared to pristine P3HT film; this complies well with absorption and XRD results.

### Proposed Energy Level and Exciton Dynamics Model of P3HT-ZnO Heterostructure

**Fig. 10:**
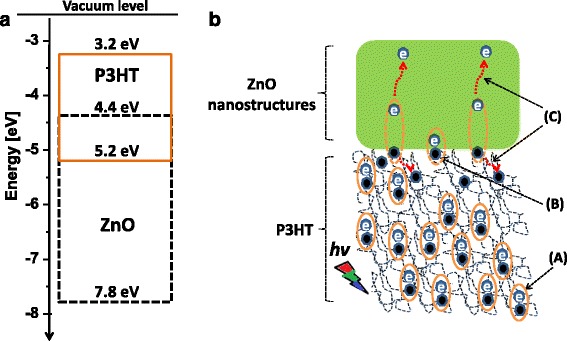
Schematic sketch of **a** energy level and **b** exciton dynamic of a P3HT-ZnO hybrid heterostructure. (**(A)** Electron-hole generation, **(B)** interface charge separation, and **(C)** current generation)

Figure 10 depicts energy level diagram and the proposed exciton dynamic mechanism of the P3HT-ZnO hybrid heterostructure. The energy diagram (Fig. 10a) presents the *E*_LUMO_ and *E*_HOMO_, which are the energy levels within the polymer. P3HT exhibits a lowest unoccupied molecular orbital (LUMO) at about −3.2 eV while the highest unoccupied molecular orbital (HOMO) is located at −5.2 eV, both are located below the vacuum level (0 eV) [15]. Conversely, the ZnO valence band energy level *E*_V_ is situated at about −7.8 eV and the conduction band energy level at about −4.4 eV [15]. Prior to an efficient exciton dynamic process (Fig. 10b), it is of pivotal importance to achieve percolated network in the polymer in order to minimize phase separation on the macroscopic scale [14]. Upon exposure to sunlight, the donor (i.e., pristine P3HT) absorbs photons to generate electron-hole pairs (process A). Then, the diffusion of the excitons shall take place at the vicinity of the P3HT-ZnO nanostructures interface to allow charge separation (process B). Finally, the dissociated carriers (i.e., electrons and holes) are transported via the donor (polymer) and acceptor (ZnO nanostructures) to their respective electrodes and hence induce a photocurrent which relies on the energy difference between the LUMO of P3HT and the conduction band of ZnO [14]. It is however critical to take note of the subsequent competing phenomena taking place, while free carriers are formed, such as non-radiative recombination at the P3HT-ZnO interface [75]. Based on the UV-Vis and PL results, it is evident that the P3HT-ZnO:Ho^3+^ film more likely exhibit an enhanced charge mobility relative to the pristine P3HT film.

## Conclusions

The optoelectronic and microstructural properties of P3HT-ZnO:Ho^3+^ heterostructure film has been successfully investigated by XRD, UV-Vis, PL, and Raman spectroscopy. ZnO:Ho^3+^ nanostructures were found to be effective PL quencher agent in the P3HT polymer. Strong enhancement and slight redshift of P3HT absorbance spectrum were obtained as a result of inclusion of selective amount of ZnO:Ho^3+^ nanostructures. In addition to the PL quenching, these findings indicate that ZnO:Ho^3+^ nanostructure inclusion to the P3HT matrix improved the crystal quality and optoelectronic properties, among which the optical conductivity. The current study revealed that Ho^3+^ played a dramatic role in improving the quality of the heterojunction interface and charge transfer within the heterostructure-based P3HT polymer. Overall, P3HT-ZnO:Ho^3+^ heterostructures appear to be a promising candidate for application in various optoelectronic devices. In order to effectively confirm the effect of Ho^3+^ on the charge transfer dynamics properties of P3HT-ZnO:Ho^3+^ heterostructure for better device application, we are currently investigating the excited state fluorescence lifetime.
